# Prepregnancy body mass index in Ontario from 2012 to 2022: A population-based descriptive study

**DOI:** 10.17269/s41997-026-01214-w

**Published:** 2026-06-05

**Authors:** Sahar Khademioore, Elizabeth K. Darling, Rohan DʼSouza, Giulia M. Muraca, Yanfang Guo, Laura N. Anderson

**Affiliations:** 1 Department of Health Research Methods, Evidence and Impact, McMaster University, Hamilton, ON Canada; 2 McMaster Midwifery Research Centre, McMaster University, Hamilton, ON Canada; 3 Department of Obstetrics & Gynecology, McMaster University, Hamilton, ON Canada; 4 School of Epidemiology and Public Health, University of Ottawa, Ottawa, ON Canada; 5 Better Outcomes Registry and Network (BORN) Ontario, Childrenʼs Hospital of Eastern Ontario, Ottawa, ON Canada

**Keywords:** Body mass index, Pregnancy, Socioeconomic factors, Obesity, Epidemiology, Indice de masse corporelle, Grossesse, Facteurs socioéconomiques, Obésité, Épidémiologie

## Abstract

**Objectives:**

To examine temporal trends in prepregnancy body mass index (BMI) from 2012 to 2022 in Ontario, Canada, and identify disparities across sociodemographic and medical condition subgroups.

**Methods:**

We conducted a population-based descriptive study using data from the Better Outcomes Registry & Network (BORN) Ontario. The study included 1,170,997 singleton births between April 2012 and March 2022. Prepregnancy BMI was categorized according to World Health Organization classifications. We used generalized estimating equations to assess temporal trends in mean BMI and BMI categories, with sequential adjustments for socioeconomic and medical factors. Analyses were stratified by maternal age, parity, race/ethnicity, neighbourhood income, education, and material deprivation quantiles, smoking status, and pre-existing medical conditions.

**Results:**

Mean prepregnancy BMI remained stable at 25.3 kg/m^2^ from 2012 to 2016, then increased to 26.0 kg/m^2^ by 2021. From 2012 to 2022, the proportion of individuals with underweight or normal weight BMI decreased (underweight 8.6% to 6.7%; normal weight 50.2% to 46.0%), while overweight and all obesity classes increased (overweight 20.4% to 22.4%; obesity class I 13.5% to 16.2%; obesity class II 3.7% to 4.6%; obesity class III 3.6% to 4.2%). After full adjustment, average BMI increased by 0.098 kg/m^2^ (95% CI 0.094–0.101) annually. An inverse socioeconomic gradient was evident across neighbourhood education and income, and material deprivation quintiles, with individuals in lower quintiles exhibiting higher prepregnancy BMI compared with those in higher quintiles.

**Conclusion:**

Prepregnancy BMI increased significantly in Ontario between 2012 and 2022, with acceleration from 2016 onward. Marked disparities across sociodemographic subgroups highlight the need for targeted preconception health interventions.

## Résumé

**Objectifs:**

Étudier les tendances temporelles de l’indice de masse corporelle (IMC) de pré-grossesse entre 2012 et 2022 en Ontario, au Canada, et cerner les disparités entre les sous-groupes sociodémographiques et médicaux.

**Méthodes:**

Nous avons mené une étude populationnelle descriptive à l’aide des données du Registre et Réseau des Bons Résultats dès la naissance (BORN) de l’Ontario. L’étude a porté sur 1 170 997 naissances uniques survenues entre avril 2012 et mars 2022. L’IMC de pré-grossesse était catégorisé selon la classification de l’Organisation mondiale de la santé. Nous avons utilisé des équations d’estimation généralisées pour évaluer les tendances temporelles de l’IMC moyen et des catégories d’IMC, avec des ajustements séquentiels pour tenir compte des facteurs socioéconomiques et médicaux. Les analyses ont été stratifiées selon l’âge maternel, la parité, la race/l’origine ethnique, le revenu du quartier, le niveau d’instruction, les quantiles de défavorisation matérielle, l’usage du tabac et les troubles médicaux préexistants.

**Résultats:**

L’IMC de pré-grossesse moyen est resté stable, à 25,3 kg/m^2^, de 2012 à 2016, puis il a grimpé à 26 kg/m^2^ en 2021. De 2012 à 2022, la proportion des sujets ayant un IMC insuffisant ou normal a diminué (poids insuffisant: de 8,6% à 6,7%; poids normal: de 50,2% à 46%), tandis que le surpoids et toutes les catégories d’obésité ont augmenté (surpoids: de 20,4% à 22,4%; catégorie d’obésité I: de 13,5% à 16,2%; catégorie d’obésité II: de 3,7% à 4,6%; catégorie d’obésité III: de 3,6% à 4,2%). Après tous les ajustements, l’IMC moyen a augmenté de 0,098 kg/m^2^ (IC de 95%: 0,094–0,101) annuellement. Un gradient socioéconomique inverse était manifeste pour tous les niveaux d’instruction et de revenu du quartier et pour tous les quintiles de défavorisation matérielle, les sujets des quintiles inférieurs présentant un IMC de pré-grossesse plus élevé que les sujets des quintiles supérieurs.

**Conclusion:**

L’IMC de pré-grossesse a présenté une augmentation significative en Ontario entre 2012 et 2022, avec une accélération à partir de 2016. Les disparités marquées d’un sous-groupe sociodémographique à l’autre soulignent le besoin d’interventions sanitaires ciblées avant la conception.

## Introduction

Obesity rates have risen worldwide over the past decades (Blüher, 2019; Phelps et al., 2024). This upward trend accelerated during the COVID-19 pandemic (Anderson et al., 2023). In Canada, the prevalence of obesity increased from 25% in 2009 to 33% in 2023, with the annual increase in obesity doubling during the pandemic period. Notably, women experienced disproportionate increases, with a 9% increase in obesity among females compared to 7% in males, and young adults were particularly impacted (Anderson et al., 2025). These accelerated trends raise significant concerns about population-level implications for pregnancy outcomes.

Body mass index (BMI) is commonly used to classify weight status, with obesity defined as BMI ≥ 30 kg/m^2^. While BMI has limitations as a measure of individual health as not all individuals with BMI ≥ 30 have adverse metabolic profiles (Adab et al., 2018), it remains a recommended tool for population-level health screening and risk stratification (Carter et al., 2025; Wharton et al., 2020). Maternal obesity is associated with higher risk of adverse outcomes such as gestational diabetes, hypertensive disorders, postpartum haemorrhage, and preterm premature rupture of membrane (Vats et al., 2021). Conversely, maternal underweight is associated with small for gestational age infant and preterm birth (Vats et al., 2021).

Comprehensive surveillance data examining temporal patterns of prepregnancy BMI remain limited. Current research on prepregnancy BMI trends has been predominantly conducted in the United States (Simpson et al., 2024; Wang et al., 2021), with only one study containing data spanning the COVID-19 pandemic period, which reported an increase in prepregnancy obesity (Simpson et al., 2024). While international studies have documented concerning trends in countries such as Brazil, where prepregnancy obesity increased from 9.8% to 19.8% between 2008 and 2018 (Carrilho et al., 2022), Canadian-specific data encompassing the pandemic period remain absent from the literature.

Therefore, the objective of this study was to examine prepregnancy BMI trends from 2012 to 2022 in Ontario, Canada. We stratified analyses by key sociodemographic characteristics to identify potential disparities and inform targeted public health strategies for improving preconception health and pregnancy outcomes.

## Methods

### Study design and data sources

This study was conducted in accordance with the framework for descriptive epidemiology proposed by Lesko et al. (2022). We conducted a population-based descriptive study using data obtained from the Better Outcomes Registry & Network (BORN) Ontario (Murphy et al., 2021). BORN Ontario operates as a prescribed registry under Ontario’s Personal Health Information Protection Act (PHIPA). The BORN Information System (BIS) provides comprehensive perinatal data coverage, capturing nearly all births across the province, with approximately 140,000 births recorded each year, representing about 40% of all births in Canada (Dunn et al., 2019; Murphy et al., 2021).

Canadian Census 2021 data, accessed using Statistics Canada’s Postal Code Conversion File Plus, were linked to maternal residential postal codes at the dissemination area level to obtain neighbourhood-level socioeconomic status indicators. This study received ethics approval from the Hamilton Integrated Research Ethics Board (Project ID: 16828).

### Study population

We included pregnant individuals with a singleton live birth or stillbirth, in a hospital setting in Ontario, Canada, between April 1, 2012, and March 31, 2022. Eligible births were those ≥ 20 weeks of gestation or with a birthweight greater than 500 g. Individuals with missing data on prepregnancy BMI were excluded, as prepregnancy BMI is the primary variable of interest and individuals with missing prepregnancy BMI could not contribute to the analysis. To reduce the impact of biologically implausible or extreme outliers, we excluded records with prepregnancy BMI values below 15 or above 70 (Lebel et al., 2018). These exclusion criteria are consistent with prior studies (Guo et al., 2019; Palumbo et al., 2025).

### Measurement of prepregnancy BMI

Prepregnancy BMI was calculated by dividing prepregnancy weight (kg) by the square of height (m^2^). In the BIS, prepregnancy weight was obtained either from the weight recorded during Multiple Marker Screening (first trimester) minus 2 kg, or from self-reported prepregnancy weight at delivery (Dayan et al., 2018; Srugo et al., 2021). BMI was categorized according to World Health Organization (WHO) classifications: underweight (< 18.5), normal weight (18.5–24.9), overweight (25.0–29.9), obesity class I (30.0–34.9), obesity class II (35.0–39.9), and obesity class III (≥ 40.0).

### Other variables

The primary independent variable of interest was the year of birth, defined according to the infant’s date of birth and categorized by fiscal year from April 2012 to March 2022, consistent with the administrative structure of BORN Ontario data.

We identified a list of covariates based on previous literature to account for demographic, socioeconomic, and medical characteristics known to be associated with prepregnancy BMI, as well as to examine subgroup differences over time (Cheng et al., 2022; Poston et al., 2016). These covariates included maternal age, parity, race/ethnicity, median neighbourhood household income quintile, neighbourhood education quintile, material deprivation quintile, prepregnancy smoking status, a composite measure of prepregnancy medical conditions, and diabetes status. Race/ethnicity data were obtained from prenatal screening requisitions, either self-reported by pregnant individuals or recorded by healthcare providers. Median neighbourhood income represents the neighbourhood income distribution. Neighbourhood education quintile reflects the proportion of adults in the neighbourhood with post-secondary education, with higher quintiles indicating greater educational attainment, while the material deprivation quintile reflected an area-level deprivation index incorporating factors such as employment, housing quality, and access to resources. The composite medical condition variable included prepregnancy chronic hypertension, cardiovascular disease, asthma, and pulmonary disease. Race/ethnicity was included in the models to account for known racial and ethnic disparities in BMI (Simpson et al., 2024).

### Statistical analyses

We visualized the temporal trend in prepregnancy BMI by plotting mean BMI and proportions of BMI categories with 95% confidence intervals (CIs), in each fiscal year, stratified by study covariates.

We fitted regression models to assess temporal trends in prepregnancy BMI. For mean BMI, we applied generalized estimating equations (GEE) with a normal distribution, identity link, and exchangeable working correlation structure to estimate beta coefficients. For BMI categories, we used GEE with a multinomial distribution, logit link, and independent working correlation structure to estimate odds ratios. GEE was selected to account for within-person correlation arising from individuals contributing multiple pregnancies over the study period. Within the GEE models, we performed sequential adjustments in three steps: (1) unadjusted models; (2) models adjusted for socioeconomic factors (age, parity, race/ethnicity, median neighbourhood household income, neighbourhood education quintile, material deprivation quintile); and (3) fully adjusted models including medical factors (prepregnancy diabetes, pre-existing medical conditions, and smoking status). This stepwise approach helped evaluate how each group of covariates contributed to temporal changes in prepregnancy BMI.

Separately, temporal trends in mean BMI were analyzed using Joinpoint regression (Surveillance Research Program, 2025), which identified significant inflection points based on Bayesian Information Criterion (BIC) model selection (*α* = 0.05).

Multiple imputation (MI) was used to impute missing data for covariates in SAS. The imputation model included all analyzed variables; prepregnancy BMI category and study year were included as predictors in the MI models but were not imputed. All covariates with missing values were categorical and imputed using the discriminant function method assuming that missing values were missing at random. Five imputed datasets were generated using PROC MI, and parameter estimates and standard errors were pooled across imputations using PROC MIANALYZE according to  Rubin’s rules.

There was < 5% missing for all covariates (pre-existing medical conditions 3.5%; income quintile 2.8%; deprivation quintile 2.7%; education quintile 2.3%; smoking status 1.9%; race/ethnicity 1.0%; prepregnancy diabetes 0.9%; parity 0.6%; maternal age < 0.1%). We treated missing race/ethnicity data as a separate category to differentiate individuals who did not undergo prenatal screening from those who completed screening but had missing information. All analyses were performed in SAS version 9.4 (SAS Institute, Cary, North Carolina) or R version 4.5.1 (R Foundation for Statistical Computing, Vienna, Austria).

### Sensitivity analysis

We also modeled year as a categorical variable to capture year-specific variations in prepregnancy BMI. To evaluate potential selection bias, we compared individuals excluded due to missing BMI data with the study cohort using standardized mean differences (SMD).

## Results

### Population characteristics

**Fig. 1:**
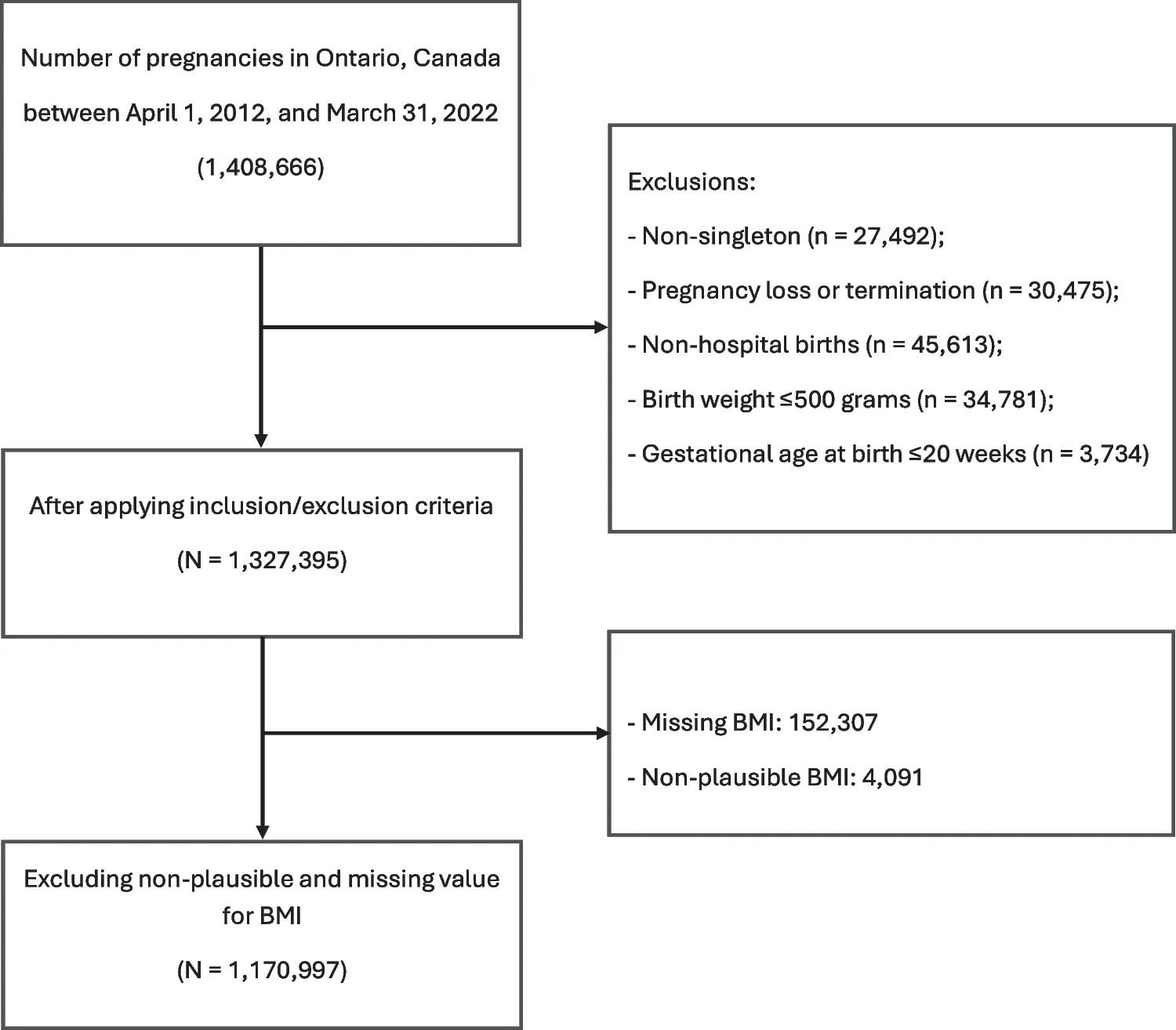
Flow diagram of study population after applying inclusion criteria (Appendix A)

Between April 2012 and March 2022, there were 1,408,666 live births recorded in the BORN Ontario dataset. After applying inclusion criteria and excluding records with missing prepregnancy BMI data (11.8%), the final analytic cohort comprised 1,170,997 births (Fig. 1, Appendix A). Table 1 summarizes the demographic, clinical, and socioeconomic characteristics of the study population across the study period.

**Table 1:** Characteristics of the singleton births in Ontario, Canada, from 2012 to 2022, *N* = 1,170,997

Characteristic	2012–2013	2013–2014	2014–2015	2015–2016	2016–2017	2017–2018	2018–2019	2019–2020	2020–2021	2021–2022	All years
Maternal age (years)											
< 18	924 (0.9)	794 (0.7)	749 (0.6)	679 (0.6)	574 (0.5)	482 (0.4)	386 (0.3)	370 (0.3)	331 (0.3)	263 (0.2)	5552 (0.5)
18–24	15,038 (14.0)	15,539 (13.7)	15,566 (13.5)	14,883 (12.5)	14,327 (11.6)	13,286 (10.9)	12,298 (10.2)	11,454 (9.6)	10,343 (9.0)	9016 (7.8)	131,750 (11.3)
25–29	30,216 (28.1)	31,753 (28.0)	32,326 (28.0)	32,791 (27.6)	33,414 (27.1)	32,391 (26.6)	31,447 (26.2)	31,068 (26.0)	29,320 (25.5)	28,106 (24.2)	312,832 (26.7)
30–34	37,746 (35.1)	40,536 (35.8)	41,514 (36.0)	43,494 (36.6)	46,032 (37.3)	45,539 (37.4)	45,450 (37.8)	46,027 (38.5)	45,250 (39.3)	46,797 (40.3)	438,385 (37.4)
35–39	19,345 (18.0)	20,180 (17.8)	20,735 (18.0)	22,278 (18.7)	23,764 (19.3)	24,530 (20.2)	25,182 (21.0)	25,115 (21.0)	24,625 (21.4)	26,343 (22.7)	232,097 (19.8)
≥ 40	4188 (3.9)	4548 (4.0)	4483 (3.9)	4818 (4.1)	5095 (4.1)	5236 (4.3)	5289 (4.4)	5531 (4.6)	5162 (4.5)	5524 (4.8)	49,874 (4.3)
Missing			< 6 (S)		122 (0.1)	250 (0.2)	125 (0.1)	< 6 (S)	< 6 (S)	< 6 (S)	507 (0.0)
Median household income quintile											
Q1 (lowest)	23,060 (21.5)	24,201 (21.4)	24,925 (21.6)	25,043 (21.1)	25,861 (21.0)	25,541 (21.0)	24,631 (20.5)	24,370 (20.4)	22,967 (20.0)	22,109 (19.1)	242,708 (20.7)
Q2	19,897 (18.5)	20,834 (18.4)	21,480 (18.6)	22,217 (18.7)	22,832 (18.5)	22,796 (18.7)	22,622 (18.8)	22,502 (18.8)	21,508 (18.7)	21,469 (18.5)	218,157 (18.6)
Q3	18,992 (17.7)	20,229 (17.8)	20,798 (18.0)	21,272 (17.9)	22,104 (17.9)	21,898 (18.0)	21,657 (18.0)	22,019 (18.4)	21,047 (18.3)	21,712 (18.7)	211,728 (18.1)
Q4	21,624 (20.1)	22,597 (19.9)	22,932 (19.9)	24,429 (20.5)	25,293 (20.5)	25,064 (20.6)	25,240 (21.0)	25,228 (21.1)	24,619 (21.4)	25,299 (21.8)	242,325 (20.7)
Q5 (highest)	21,345 (19.9)	22,117 (19.5)	21,771 (18.9)	22,430 (18.9)	23,629 (19.2)	22,913 (18.8)	22,641 (18.8)	22,046 (18.4)	21,767 (18.9)	22,299 (19.2)	222,958 (19.0)
Missing	2539 (2.4)	3372 (3.0)	3469 (3.0)	3552 (3.0)	3609 (2.9)	3502 (2.9)	3386 (2.8)	3401 (2.8)	3126 (2.7)	3165 (2.7)	33,121 (2.8)
Education quintiles at dissemination level											
Q1 (lowest)	21,315 (19.8)	22,368 (19.7)	23,035 (20.0)	23,148 (19.5)	23,525 (19.1)	23,047 (18.9)	22,700 (18.9)	22,137 (18.5)	21,016 (18.3)	20,812 (17.9)	223,103 (19.1)
Q2	22,059 (20.5)	23,436 (20.7)	24,214 (21.0)	24,520 (20.6)	24,899 (20.2)	24,768 (20.3)	24,459 (20.4)	24,826 (20.8)	23,436 (20.4)	23,581 (20.3)	240,198 (20.5)
Q3	19,334 (18.0)	20,197 (17.8)	20,310 (17.6)	20,981 (17.6)	21,596 (17.5)	21,743 (17.9)	21,213 (17.7)	21,170 (17.7)	20,713 (18.0)	20,763 (17.9)	208,020 (17.8)
Q4	23,507 (21.9)	24,559 (21.7)	25,464 (22.1)	26,598 (22.4)	27,698 (22.5)	27,472 (22.6)	27,218 (22.6)	27,308 (22.8)	26,765 (23.3)	27,259 (23.5)	263,848 (22.5)
Q5 (highest)	19,344 (18.0)	20,115 (17.7)	19,513 (16.9)	20,864 (17.5)	22,724 (18.4)	21,880 (18.0)	21,866 (18.2)	21,370 (17.9)	20,621 (17.9)	21,088 (18.2)	209,385 (17.9)
Missing	1898 (1.8)	2675 (2.4)	2839 (2.5)	2832 (2.4)	2886 (2.3)	2804 (2.3)	2721 (2.3)	2755 (2.3)	2483 (2.2)	2550 (2.2)	26,443 (2.3)
Material deprivation quintiles											
Q1 (least)	17,587 (16.4)	18,796 (16.6)	18,814 (16.3)	19,798 (16.6)	21,103 (17.1)	20,946 (17.2)	21,087 (17.5)	21,122 (17.7)	20,924 (18.2)	22,294 (19.2)	202,471 (17.3)
Q2	21,326 (19.8)	22,686 (20.0)	22,993 (19.9)	24,208 (20.4)	25,203 (20.4)	24,941 (20.5)	25,028 (20.8)	24,725 (20.7)	24,375 (21.2)	25,015 (21.6)	240,500 (20.5)
Q3	21,685 (20.2)	22,583 (19.9)	23,069 (20.0)	23,851 (20.1)	24,708 (20.0)	23,945 (19.7)	23,610 (19.6)	23,743 (19.9)	22,779 (19.8)	22,955 (19.8)	232,928 (19.9)
Q4	19,878 (18.5)	20,591 (18.2)	21,105 (18.3)	21,428 (18.0)	22,156 (18.0)	22,173 (18.2)	21,660 (18.0)	21,790 (18.2)	20,706 (18.0)	20,770 (17.9)	212,257 (18.1)
Q5 (most)	24,523 (22.8)	25,440 (22.4)	25,976 (22.5)	26,223 (22.0)	26,691 (21.6)	26,341 (21.6)	25,467 (21.2)	24,831 (20.8)	23,257 (20.2)	21,986 (18.9)	250,735 (21.4)
Missing	2458 (2.3)	3254 (2.9)	3418 (3.0)	3435 (2.9)	3467 (2.8)	3368 (2.8)	3325 (2.8)	3355 (2.8)	2993 (2.6)	3033 (2.6)	32,106 (2.7)
Race/ethnicity											
Asian	16,186 (15.1)	18,467 (16.3)	19,776 (17.1)	20,680 (17.4)	22,648 (18.4)	22,246 (18.3)	23,193 (19.3)	24,007 (20.1)	24,329 (21.1)	24,221 (20.9)	215,753 (18.4)
Black	4198 (3.9)	4778 (4.2)	4867 (4.2)	5098 (4.3)	5468 (4.4)	5453 (4.5)	5754 (4.8)	5988 (5.0)	6108 (5.3)	6074 (5.2)	53,786 (4.6)
White	36,542 (34.0)	47,338 (41.8)	47,972 (41.6)	49,792 (41.9)	51,729 (41.9)	51,389 (42.2)	51,820 (43.1)	51,575 (43.1)	50,101 (43.6)	51,776 (44.6)	490,034 (41.8)
Other^a^	3085 (2.9)	3904 (3.4)	4212 (3.7)	4689 (3.9)	5377 (4.4)	5798 (4.8)	5233 (4.4)	5157 (4.3)	5089 (4.4)	5544 (4.8)	48,088 (4.1)
Missing due to no PSO screening	45,897 (42.7)	37,042 (32.7)	36,710 (31.8)	36,451 (30.6)	35,941 (29.1)	35,587 (29.2)	33,998 (28.3)	32,688 (27.3)	29,260 (25.4)	28,290 (24.4)	351,864 (30.0)
Missing information for race/ethnicity in PSO screening	1549 (1.4)	1821 (1.6)	1838 (1.6)	2233 (1.9)	2165 (1.8)	1241 (1.0)	179 (0.1)	151 (0.1)	147 (0.1)	148 (0.1)	11,472 (1.0)
Parity											
Nulliparous (0)	46,978 (43.7)	49,875 (44.0)	50,410 (43.7)	51,544 (43.3)	52,702 (42.7)	51,991 (42.7)	51,772 (43.1)	52,828 (44.2)	51,785 (45.0)	51,835 (44.7)	511,720 (43.7)
Primiparous (1)	37,787 (35.2)	39,834 (35.1)	40,288 (34.9)	41,397 (34.8)	42,364 (34.4)	41,845 (34.4)	42,936 (35.7)	41,746 (34.9)	40,007 (34.8)	40,900 (35.2)	409,104 (34.9)
Multiparous (≥ 2)	21,426 (19.9)	22,920 (20.2)	23,011 (19.9)	25,393 (21.3)	27,643 (22.4)	27,432 (22.5)	24,950 (20.8)	24,495 (20.5)	22,923 (19.9)	23,009 (19.8)	243,202 (20.8)
Missing	1266 (1.2)	721 (0.6)	1666 (1.4)	609 (0.5)	619 (0.5)	446 (0.4)	519 (0.4)	497 (0.4)	319 (0.3)	309 (0.3)	6,971 (0.6)
Smoking status											
No	94,944 (88.4)	99,743 (88.0)	96,707 (83.8)	102,720 (86.4)	108,813 (88.2)	109,811 (90.2)	109,142 (90.8)	109,367 (91.5)	106,042 (92.2)	107,999 (93.1)	1,045,288 (89.3)
Yes	11,449 (10.7)	12,021 (10.6)	12,100 (10.5)	11,875 (10.0)	11,514 (9.3)	10,918 (9.0)	9947 (8.3)	9092 (7.6)	7990 (6.9)	7012 (6.0)	103,918 (8.9)
Unknown	1064 (1.0)	1586 (1.4)	6568 (5.7)	4348 (3.7)	3001 (2.4)	985 (0.8)	1088 (0.9)	1107 (0.9)	1002 (0.9)	1042 (0.9)	21,791 (1.9)
Prepregnancy diabetes											
No	98,254 (91.4)	103,429 (91.2)	104,436 (90.5)	109,496 (92.1)	112,809 (91.5)	110,398 (90.7)	108,324 (90.1)	106,555 (89.1)	102,886 (89.4)	102,912 (88.7)	1,059,499 (90.5)
Yes	6523 (6.1)	7075 (6.2)	8136 (7.1)	8826 (7.4)	10,265 (8.3)	11,059 (9.1)	11,595 (9.6)	12,710 (10.6)	11,931 (10.4)	12,508 (10.8)	100,628 (8.6)
Missing	2680 (2.5)	2846 (2.5)	2803 (2.4)	621 (0.5)	254 (0.2)	257 (0.2)	258 (0.2)	301 (0.3)	217 (0.2)	633 (0.5)	10,870 (0.9)
Pre-existing medical condition^b^											
No	97,374 (90.6)	102,320 (90.3)	102,296 (88.7)	107,776 (90.6)	113,073 (91.7)	111,740 (91.8)	110,143 (91.7)	109,812 (91.8)	104,987 (91.3)	108,775 (93.7)	1,068,296 (91.2)
Yes	6090 (5.7)	6421 (5.7)	5967 (5.2)	5848 (4.9)	6205 (5.0)	6006 (4.9)	6042 (5.0)	5899 (4.9)	6303 (5.5)	6615 (5.7)	61,396 (5.2)
Missing	3993 (3.7)	4609 (4.1)	7112 (6.2)	5319 (4.5)	4050 (3.3)	3968 (3.3)	3992 (3.3)	3855 (3.2)	3744 (3.3)	663 (0.6)	41,305 (3.5)

Data are presented as *n* (%) unless otherwise specified

*S*, suppressed due to small cell size (< 6);* PSO*,Prenatal Screening Ontario; *BMI*, body mass index

Prepregnancy BMI categories: underweight (< 18.5), normal weight (18.5–24.9), overweight (25.0–29.9), obesity class I (30.0–34.9), obesity class II (35.0–39.9), obesity class III (≥ 40.0)

^a^Including mixed race/ethnicity, unknown, and other groups

^b^A composite of either prepregnancy hypertension, cardiovascular disease, asthma, or pulmonary disease

**Fig. 2:**
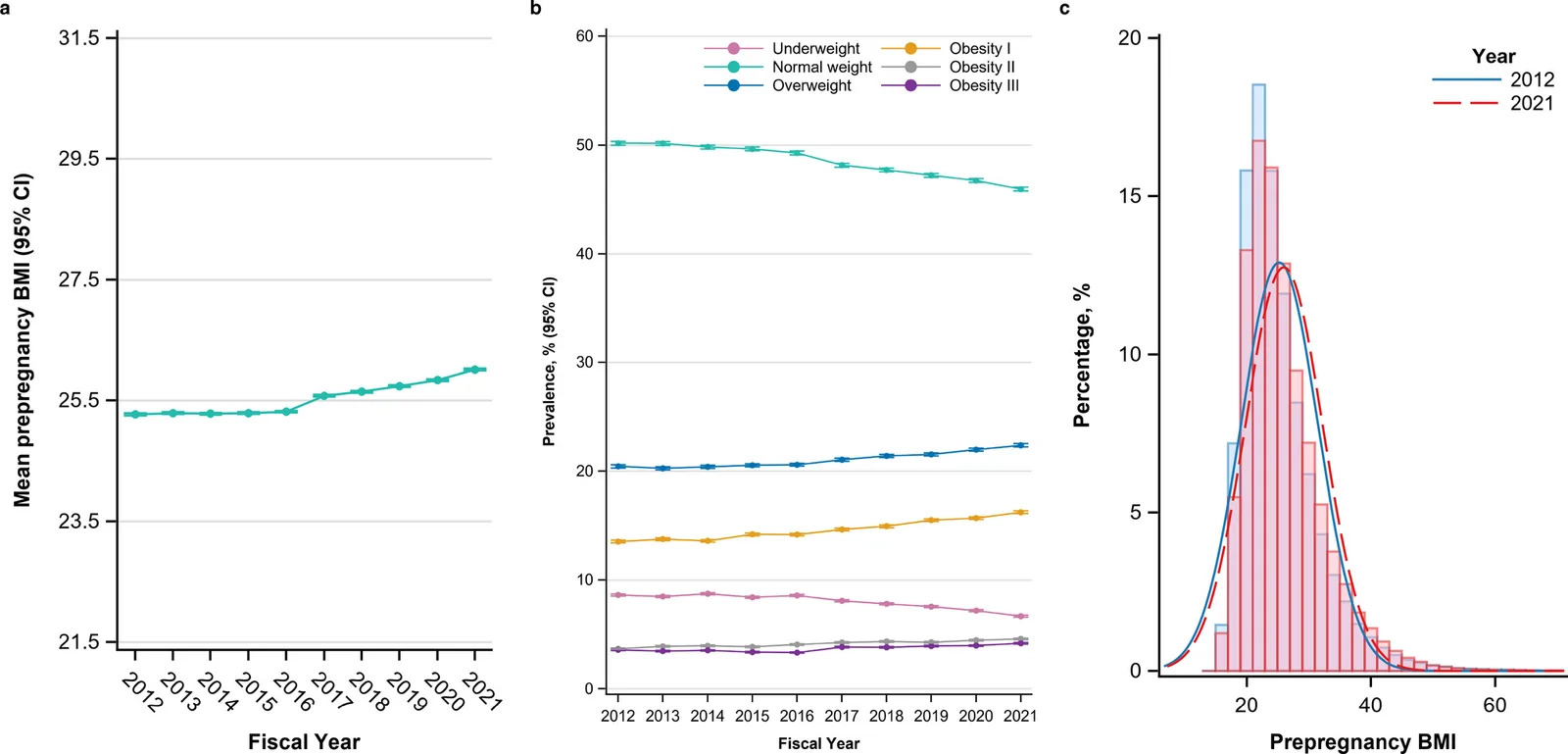
Trends in prepregnancy body mass index (BMI), Ontario 2012–2021. Annual mean prepregnancy BMI (kg/m^2^) (**a**). Annual prevalence of BMI categories (underweight, normal weight, overweight, obesity classes I–III) (**b**). Distribution of prepregnancy BMI in 2012 (blue) and 2021 (red) (**c**)

**Fig. 3:**
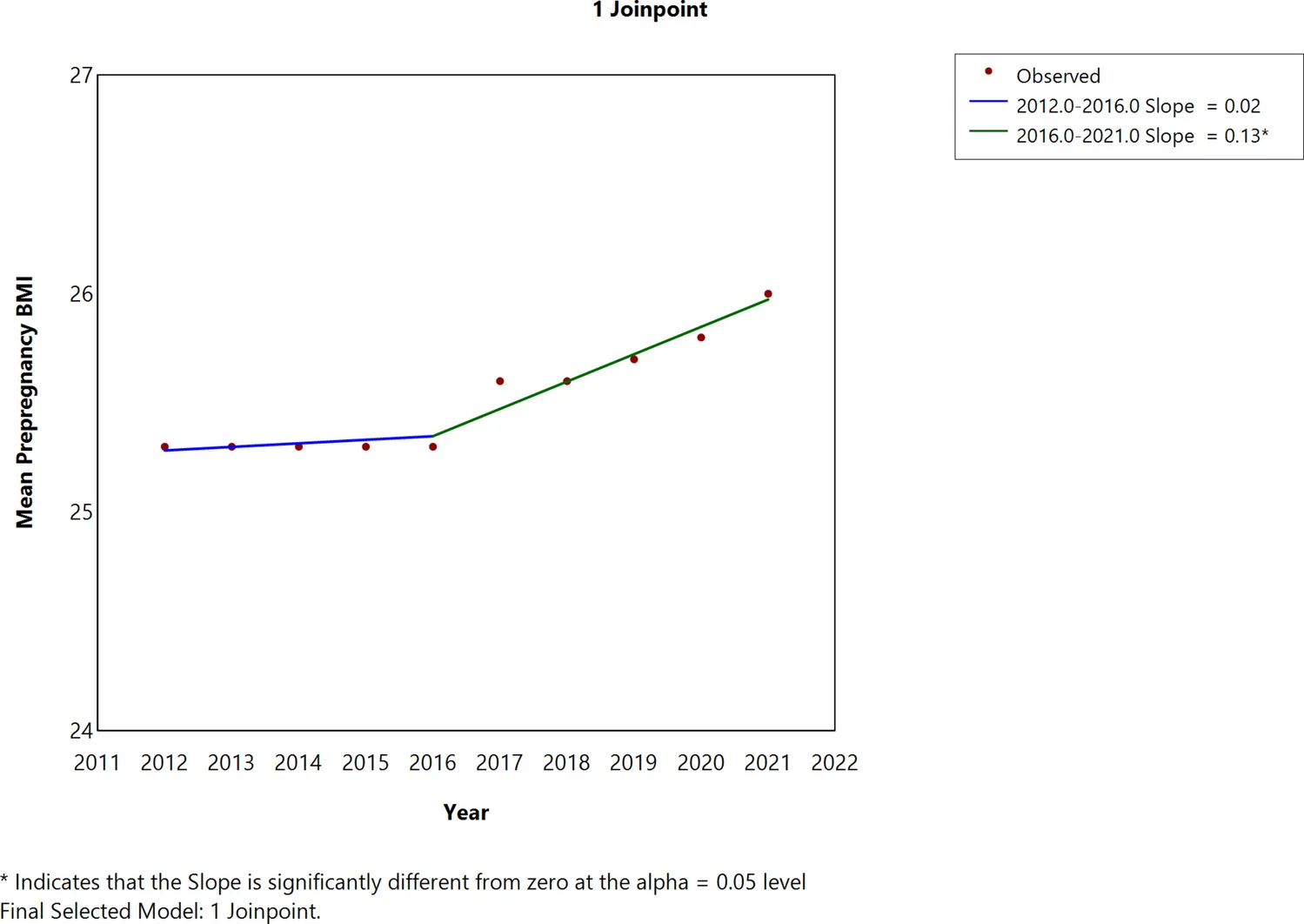
Temporal trends in mean prepregnancy BMI with joinpoint regression analysis, 2012–2021 fiscal years. Red circles represent observed mean BMI values. A significant joinpoint was identified at 2016, with BMI remaining stable from 2012 to 2016 (AAC = 0.016 units/year, *p* = 0.49) and significantly increasing from 2016 to 2021 (AAC = 0.125 units/year, *p* < 0.001)

Mean prepregnancy BMI remained stable at 25.3 kg/m^2^ (SD 6.0–6.2) from 2012 through 2016, then increased steadily to 26.0 kg/m^2^ (SD 6.3) by 2021 (Fig. 2a, and Table 3, Appendix A). The distribution of prepregnancy BMI (Fig. 2c) demonstrated a rightward population-wide shift between 2012 and 2021, with the entire distribution curve moving toward higher BMI values while maintaining a similar shape. Consistently, unadjusted linear regression models showed an annual increase of BMI by 0.205 kg/m^2^ (95% CI 0.201–0.208), which attenuated to 0.098 kg/m^2^ (95% CI 0.094–0.101) after adjustment for all covariates (Table 2). Joinpoint regression analysis identified one significant inflection point in the BMI trend at year 2016 (95% CI 2014–2019). Mean prepregnancy BMI remained stable from 2012 to 2016 (AAC = 0.016 units/year, *p* = 0.49), followed by a significant increase from 2016 to 2021 (AAC = 0.125 units/year, *p* < 0.001) (Fig. 3 and Table 4, Appendix A).

**Table 2:** Temporal trends in prepregnancy body mass index (*BMI*) in the singleton births in Ontario, Canada, from 2012 to 2022, *N* = 1,170,997

Variable	Model 1	Model 2	Model 3
Continuous mean BMI	***β* coefficient (95% CI)**	***β *coefficient (95% CI)**	***β *coefficient (95% CI)**
Per unit increase	0.205 (0.201, 0.208)	0.111 (0.107, 0.114)	0.098 (0.094, 0.101)
BMI category	**Odds ratio (95% CI)**	**Odds ratio (95% CI)**	**Odds ratio (95% CI)**
Underweight	0.984 (0.981, 0.986)	0.988 (0.985, 0.990)	0.990 (0.988, 0.993)
Normal	Reference	Reference	Reference
Overweight	1.022 (1.020, 1.023)	1.022 (1.020, 1.024)	1.020 (1.018, 1.022)
Obesity class I	1.032 (1.030, 1.034)	1.034 (1.032, 1.036)	1.030 (1.028, 1.032)
Obesity class II	1.033 (1.030, 1.037)	1.037 (1.034, 1.041)	1.031 (1.028, 1.035)
Obesity class III	1.032 (1.029, 1.036)	1.039 (1.035, 1.043)	1.031 (1.027, 1.035)

Model 1: Unadjusted

Model 2: Adjusted for maternal age, race/ethnicity, parity, and neighbourhood-level income quintile, education quintile, and material deprivation quintile

Model 3: Adjusted for maternal age, race/ethnicity, parity, and neighbourhood-level income quintile, education quintile, and material deprivation quintile, prepregnancy medical composite (prepregnancy hypertension, cardiovascular disease, asthma, or pulmonary disease), prepregnancy diabetes, and smoking status

**Fig. 4:**
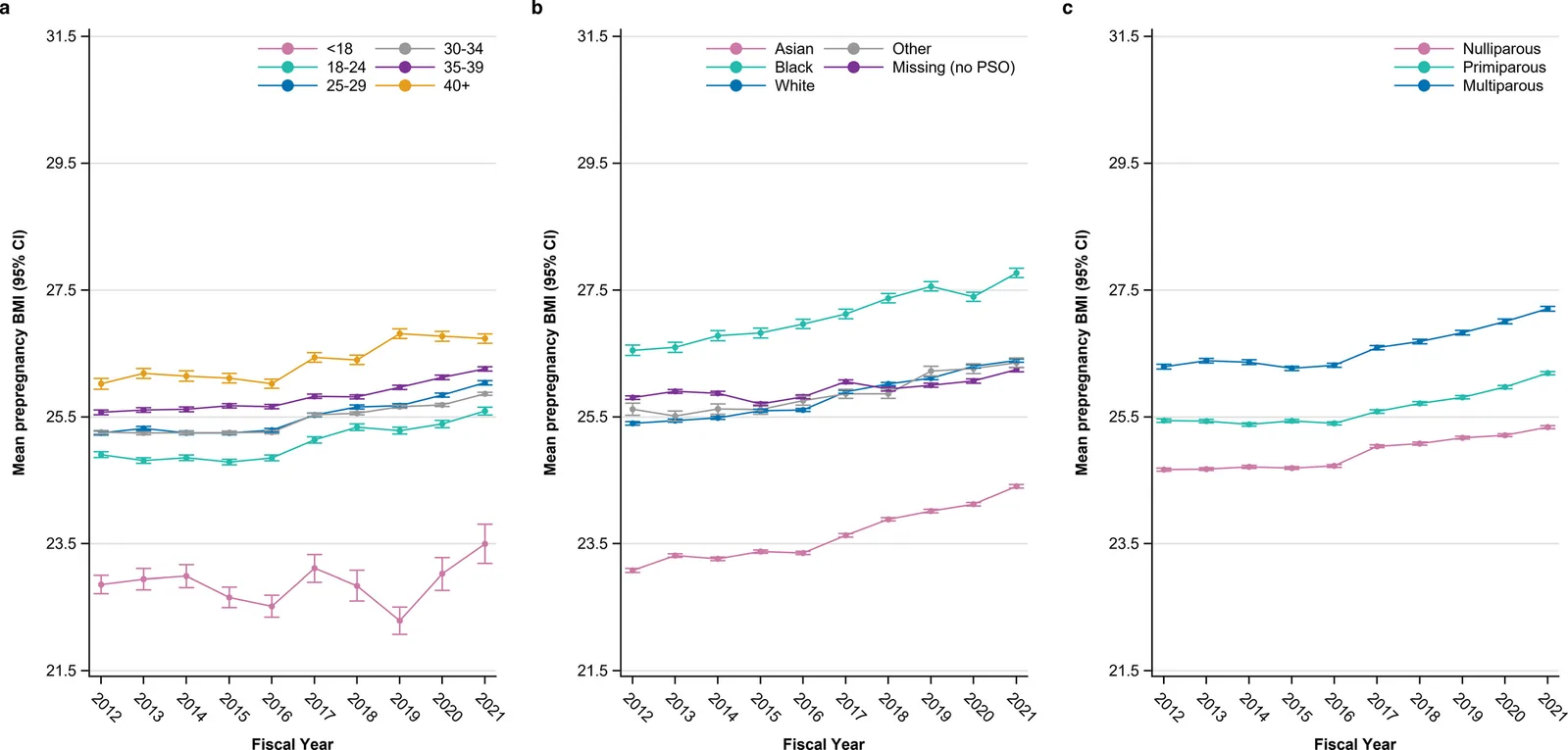
Trends in mean prepregnancy body mass index (BMI), Ontario 2012–2021, stratified by maternal age group (**a**); race/ethnicity (**b**); and parity (**c**). Data points represent annual means with error bars indicating 95% confidence intervals

**Fig. 5:**
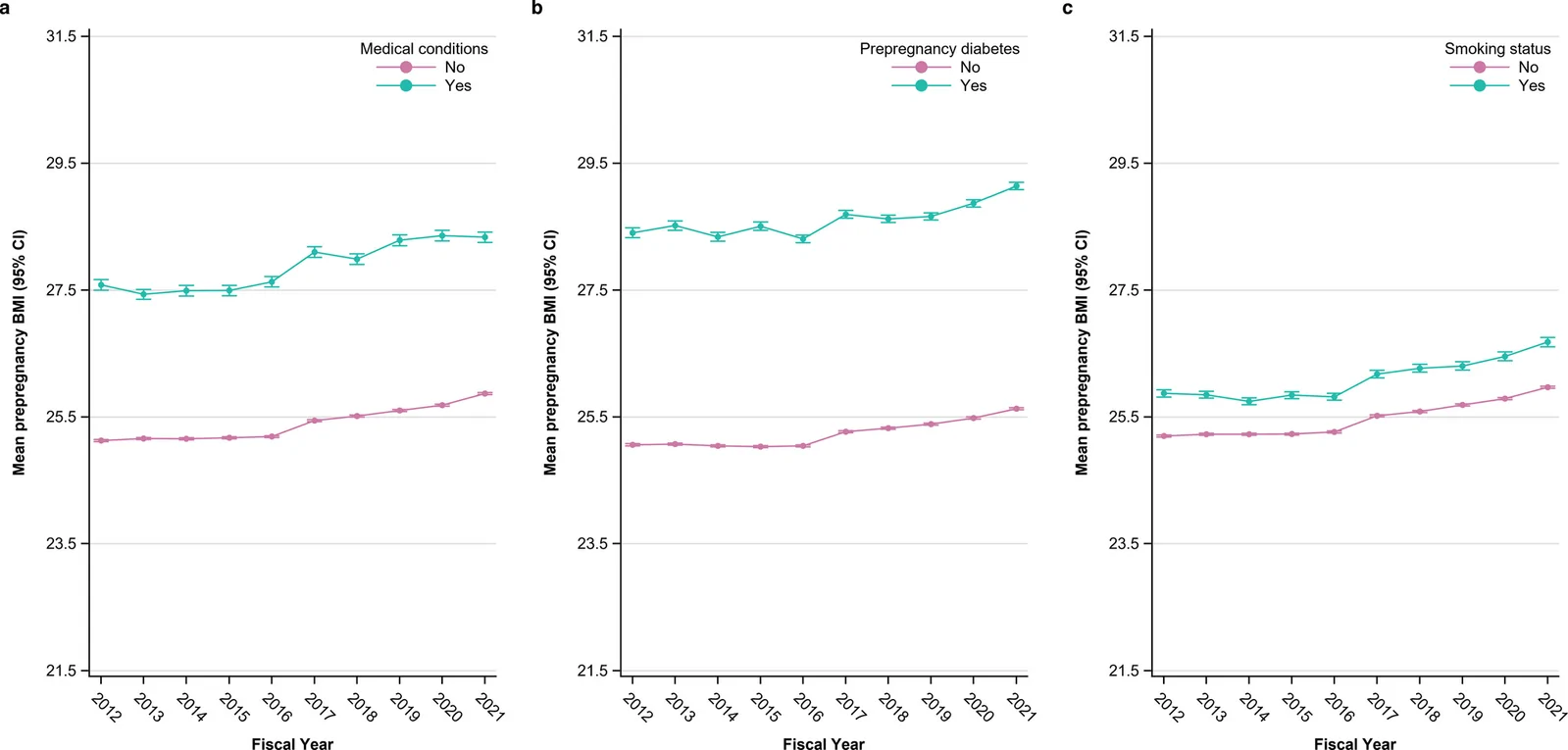
Trends in mean prepregnancy body mass index (BMI) Ontario 2012–2021 stratified by presence of prepregnancy medical condition (a composite of either prepregnancy hypertension, cardiovascular disease, asthma, or pulmonary disease) (**a**); prepregnancy diabetes status (**b**); and smoking status (**c**). Data points represent annual means with error bars indicating 95% confidence intervals

**Fig. 6:**
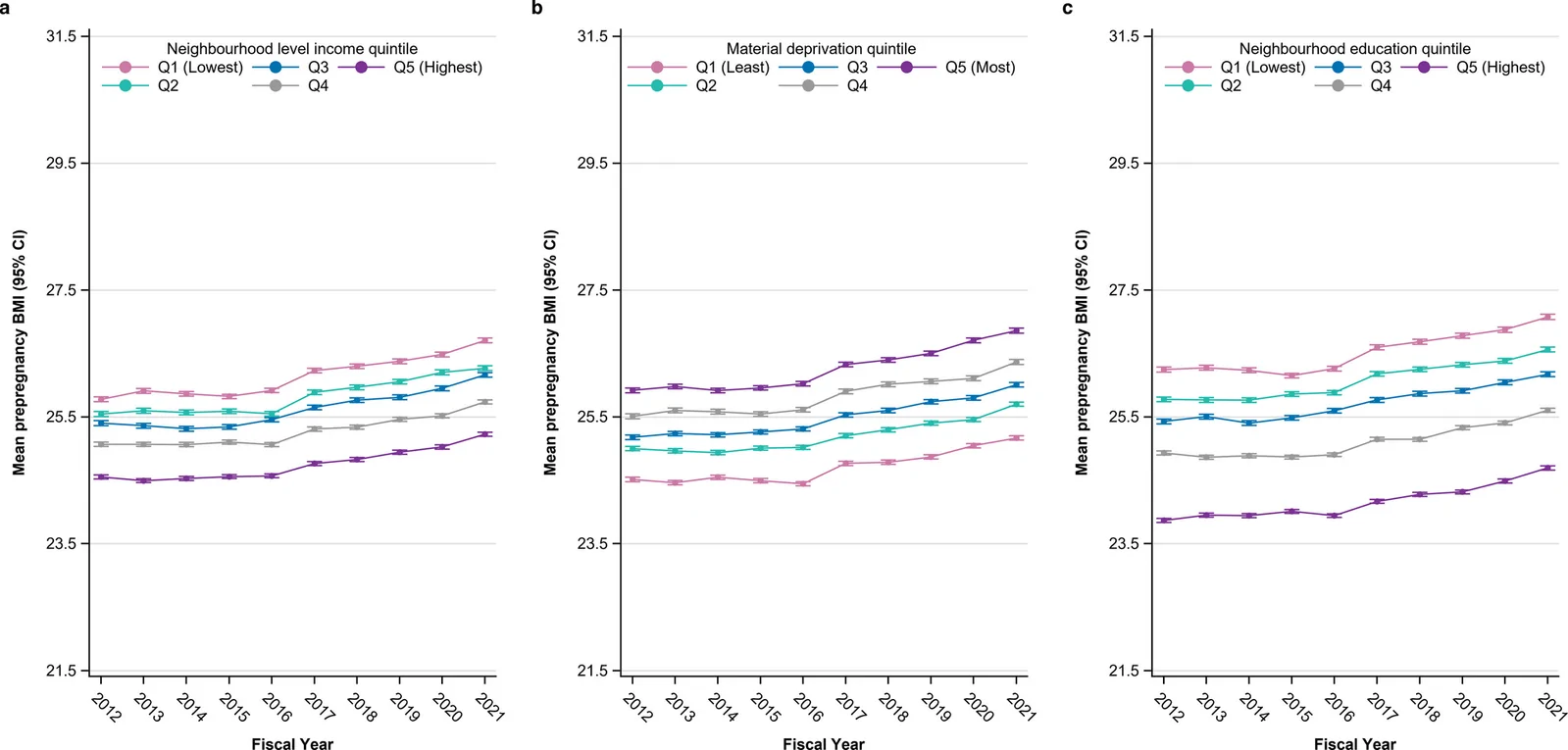
Trends in mean prepregnancy body mass index (BMI) Ontario 2012–2021 stratified by neighbourhood-level socioeconomic indicators. Neighbourhood income quintile (Q1 = lowest income, Q5 = highest income) (**a**). Neighbourhood education quintile (Q1 = lowest education attainment, Q5 = highest education attainment) (**b**). Neighbourhood material deprivation quintile (Q1 = least deprived, Q5 = most deprived) (c). Error bars represent 95% confidence intervals

We observed differences in the magnitude of prepregnancy BMI between different subgroups. Black individuals consistently had the highest mean prepregnancy BMI (26.6 to 27.8 kg/m^2^), while Asian individuals had the lowest (23.1 to 24.4 kg/m^2^) over the study period (Fig. 4b). All other race/ethnic groups had similar intermediate values (25.4–26.4 kg/m^2^). Multiparous individuals consistently had higher BMI (26.3–27.2 kg/m^2^) compared to those with one prior pregnancy (25.4–26.2 kg/m^2^) or nulliparous individuals (24.7–25.3 kg/m^2^) (Fig. 4c). Mean prepregnancy BMI increased with maternal age, with individuals aged more than 40 years having the highest BMI (26.0–26.8 kg/m^2^), while those aged less than 18 years had the lowest (22.3–23.5 kg/m^2^), and intermediate age groups showing a gradual gradient (Fig. 4a). Individuals who smoked had higher mean prepregnancy BMI compared to non-smokers (Fig. 5c). A consistent socioeconomic inverse gradient was observed across all neighbourhood indicators, with lower socioeconomic position associated with higher BMI (Fig. 6a-c). Individuals from the lowest income quintile, lowest education quintile, and most materially deprived neighbourhoods had the highest mean BMI, while those from the highest income, highest education, and least deprived neighbourhoods had the lowest BMI. Temporal patterns for pre-existing medical condition and prepregnancy diabetes are shown in Fig. 5a and b.

**Fig. 7:**
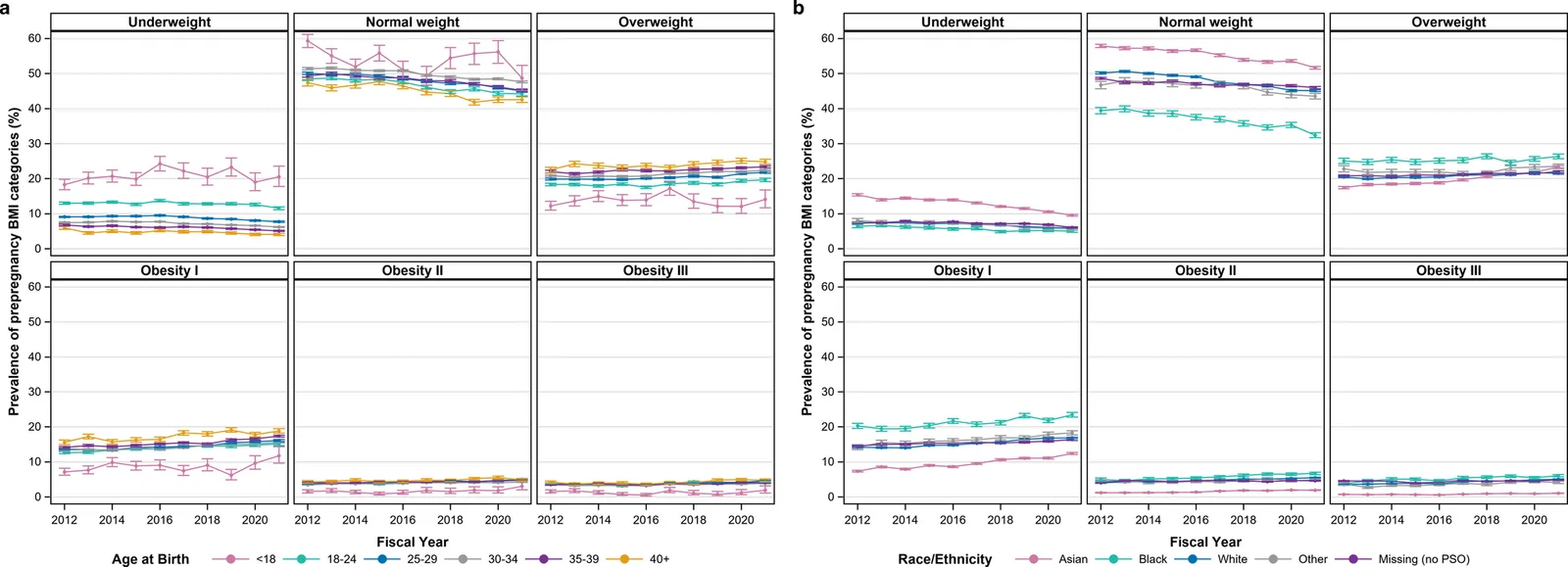
Trends in prepregnancy body mass index (BMI) categories, Ontario 2012–2021, stratified by maternal age (**a**) and race/ethnicity (**b**). Error bars represent 95% confidence intervals (Appendix A)

**Fig. 8:**
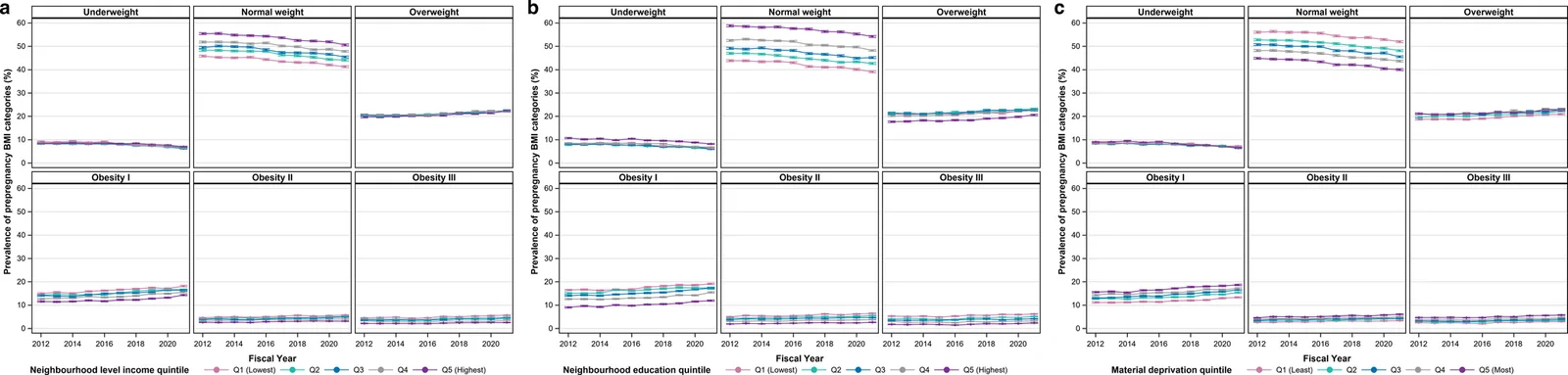
Trends in prepregnancy body mass index (BMI) categories, Ontario 2012–2021, stratified by neighborhood income quintile (Q1 = lowest income, Q5 = highest income) (**a**). Neighbourhood education quintile (Q1 = lowest education attainment, Q5 = highest education attainment) (**b**). Neighbourhood material deprivation quintile (Q1 = least deprived, Q5 = most deprived) (**c**). Error bars represent 95% confidence intervals (Appendix A)

**Fig. 9:**
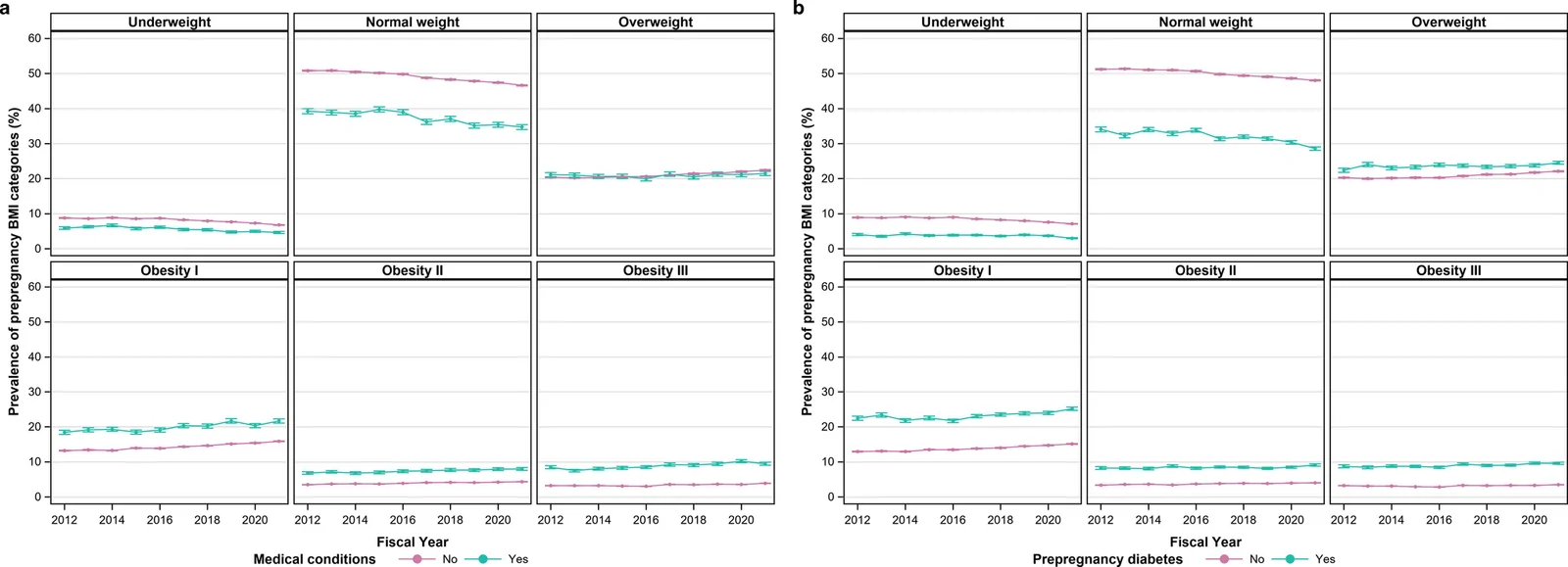
Trends in prepregnancy body mass index (BMI) categories, Ontario 2012–2021, stratified by presence of prepregnancy medical condition (a composite of either prepregnancy hypertension, cardiovascular disease, asthma, or pulmonary disease) (**a**), and prepregnancy diabetes status (**b**). Error bars represent 95% confidence intervals (Appendix A)

**Fig. 10:**
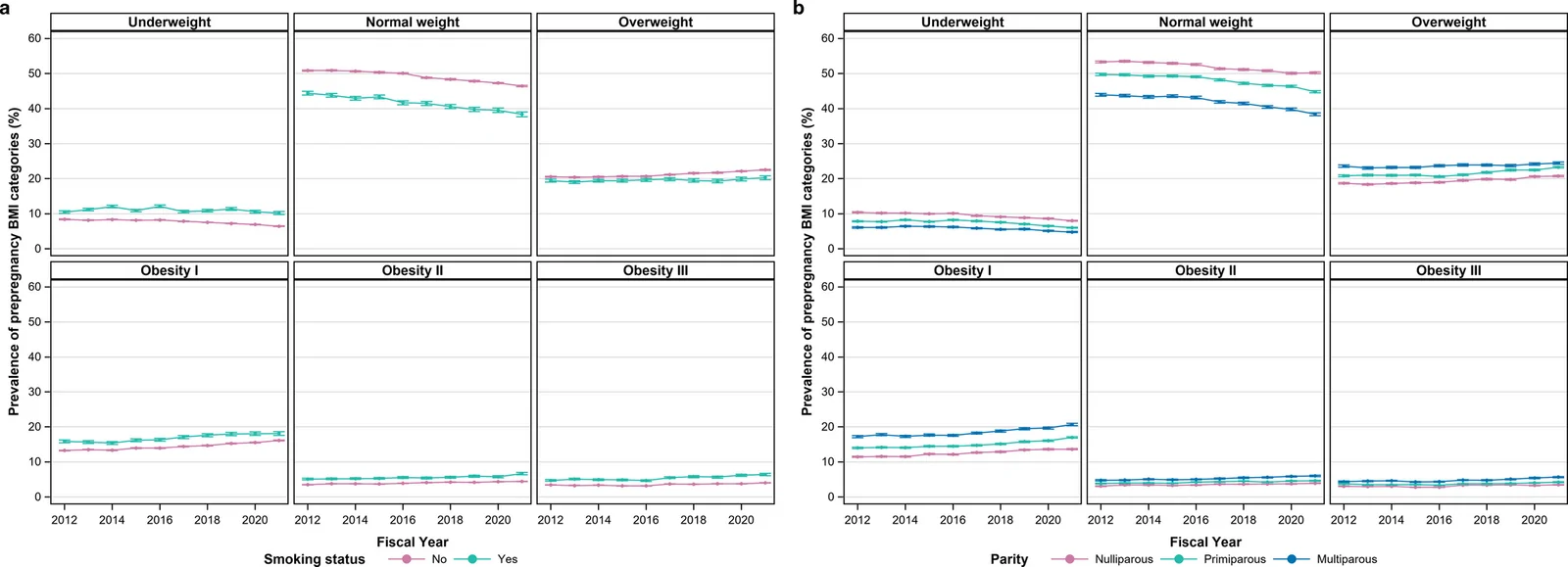
Trends in prepregnancy body mass index (BMI) categories, Ontario 2012–2021, stratified by smoking status(**a**) and parity (**b**). Error bars represent 95% confidence intervals (Appendix A)

The proportion of individuals with underweight or normal weight BMI decreased over the study period (underweight 8.6% to 6.7%; normal weight 50.2% to 46.0%), while the proportion with overweight and all obesity classes increased over the study period (overweight 20.4% to 22.4%; obesity class I 13.5% to 16.2%; obesity class II 3.7% to 4.6%; obesity class III 3.6% to 4.2%) (Fig. 2b, and Table 3, Appendix A). Multivariable regression analyses confirmed the temporal trends observed in the descriptive data (Table 2). Compared to normal weight BMI as the reference, the odds of underweight BMI decreased annually (adjusted OR 0.990, 95% CI 0.988–0.993), while the odds of overweight and all obesity classes increased over time (overweight aOR 1.020, 95% CI 1.018–1.022; obesity class I aOR 1.030, 95% CI 1.028–1.032; obesity class II aOR 1.031, 95% CI 1.028–1.035; obesity class III aOR 1.031, 95% CI 1.027–1.035). These proportions varied across subgroups. For example, individuals of Asian race/ethnicity had higher prevalence of underweight and normal weight, and lower overweight and obesity rates across all classes (Fig. 7b, Appendix A). Individuals of Black race/ethnicity had the highest overweight and obesity prevalence. BMI distribution varied by age, with younger individuals (less than 18 years) having higher rates of underweight and normal weight, and older individuals (more than 40 years) having the highest obesity and overweight prevalence (Fig. 7a, Appendix A). Additional stratifications by parity, neighbourhood education and income quintiles, material deprivation quintiles, smoking status, composite medical conditions, and prepregnancy diabetes showed varying patterns in prepregnancy BMI categories prevalence across these factors (Figs. 8, 9,10 , Appendix A).

### Sensitivity analysis

Sensitivity analyses treating year as a categorical variable were consistent with findings from models using year as continuous (Table 5, Appendix A). Mean BMI increased by 1.79 kg/m^2^ (95% CI 1.75–1.83) by 2021–2022 in unadjusted models, attenuating to 0.86 kg/m^2^ (95% CI 0.82–0.91) after adjustment, with trends accelerating from 2016 to 2017. Odds ratios showed progressive changes over time, with trends becoming more evident from 2016 to 2017 onward (Table 5). Compared with 2012–2013, unadjusted odds of underweight in 2021–2022 decreased (OR 0.85, 95% CI 0.82–0.87), while odds of overweight (OR 1.20, 95% CI 1.17–1.22) and all obesity classes increased (class I OR 1.31, 95% CI 1.28–1.34; class II OR 1.36, 95% CI 1.30–1.42; class III OR 1.28, 95% CI 1.23–1.34). Associations remained statistically significant with minimal attenuation after full covariate adjustment.

Compared to the analytic cohort (*n* = 1,170,997), participants excluded due to missing or implausible BMI (*n* = 156,398) differed primarily in the completeness of covariate data rather than in the distribution of observed covariate values. When comparing only non-missing values difference in distributions of race/ethnicity, parity, and education was observed (SMD > 0.1) (Table 6, Appendix A). However, when missing data were treated as a separate category, additional variables showed elevated SMDs (smoking status, prepregnancy diabetes and prepregnancy composite medical outcome, and material deprivation quintiles), with the differences driven predominantly by higher rates of missingness among excluded participants (Table 7, Appendix A). Additionally, earlier cohort years were overrepresented among excluded participants (SMD = 0.30), particularly 2012–2014, with distributions becoming more balanced in later years. Missingness was highest in 2012 (19.9%), declining to 7.7% by 2016 and stabilizing at approximately 10-12% thereafter (Table 6, Appendix A).

## Discussion

This population-based study of over 1.1 million pregnancies demonstrates the trend in prepregnancy BMI in Ontario between 2012 and 2022. Mean BMI remained stable from 2012 through 2016, then increased steadily through 2021, with acceleration observed from 2016 to 2017 onward. Unadjusted regression models showed annual increases in mean BMI, which attenuated after adjustment for sociodemographic and medical factors, though increasing trends persisted even after full adjustment. Examining BMI categories, the proportion of individuals with normal weight or underweight declined, while the prevalence of overweight and all obesity classes increased throughout the study period. The rightward shift of the entire prepregnancy BMI distribution indicates a population-wide phenomenon, suggesting that effective prevention requires population-based interventions rather than solely focusing on high-risk individuals (Frank & Jepson, 2013).

We observed disparities across racial/ethnic groups and socioeconomic strata, underscoring the complex interplay between social determinants of health and prepregnancy weight status. Stratification by socioeconomic characteristics is crucial for identifying and addressing health disparities (Ng et al., 2014). Obesity is shaped by complex, multilevel determinants that extend beyond individual behaviours such as nutrition and physical activity (Lee et al., 2000). Social determinants of health, including income, education, food security, neighbourhood environment, and access to healthcare, along with structural factors such as systemic racism and discriminatory policies, shape health inequities and contribute to differential obesity prevalence across population subgroups (Cockerham, 2022). Evidence demonstrates that interventions targeting obesity prevention may greatly benefit from approaches that incorporate socioeconomic factors, promoting nutrition knowledge, healthy eating, and physical activity (Sutherland et al., 2013).

Understanding temporal changes in prepregnancy BMI distributions is essential for evidence-based public health planning and resource allocation. The American Heart Association and the American College of Obstetricians and Gynecologists endorse preconception health strategies focused on improving cardiometabolic health during reproductive years, which includes appropriate weight management before pregnancy (Parikh et al., 2021). Such surveillance data can inform the development and targeting of preconception interventions, guide healthcare resource planning, and support policy initiatives aimed at improving population-based health outcomes.

Our findings are consistent with broader obesity trends observed nationally and internationally. In our study, total prepregnancy obesity prevalence increased from 20.8% in 2012 to 25.0% in 2022 (absolute increase of 4.2 percentage points). Anderson et al. (2025) reported that obesity prevalence among Canadian adults increased from 25% in 2009 to 33% in 2023, with a disproportionate increase among females, as the absolute rise in obesity prevalence between 2009 and 2023 was higher in females (8.59%) compared to males (6.93%). Wang et al. (2021) documented a 10.6% increase in prepregnancy obesity in the USA from 2013 to 2018, Simpson et al. (2024) reported continued increases in prepregnancy BMI through 2021, and Carrilho et al. (2022) found prepregnancy obesity nearly doubled in Brazil from 2008 to 2018. Our study adds to this literature by providing the first Canadian population-level data on prepregnancy BMI spanning the pandemic period with detailed examination of sociodemographic disparities.

A systematic review of 74 studies demonstrated that obesity rates increased globally during COVID-19 (Anderson et al., 2023). Mechanisms may include reduced physical activity due to lockdowns, increased stress and emotional eating, disrupted routines, and increased processed food consumption (Aghili et al., 2021). While our study was not specifically focused on evaluating COVID-19 pandemic effects, the acceleration from 2016 onward, continuing through pandemic years, suggests that contributing factors may have been building over time, with the pandemic potentially exacerbating pre-existing trajectories. Cohort replacement offers one plausible explanation for the 2016/2017 inflection. Individuals pregnant around 2016 were born in the early-to-mid 1980s and grew up during the peak of Canada’s childhood obesity epidemic, when obesity rates among children tripled between 1981 and 1996 (Spurgeon, 2002), and childhood obesity is known to track into adulthood (Simmonds et al., 2016). Importantly, the upward trend persisted after full adjustment for sociodemographic and medical factors, indicating that shifts in the compositional characteristics of the pregnant population might not account for this acceleration and that broader structural or environmental drivers are also likely contributors. The specific factors contributing to the 2016/2017 inflection point remain unclear and warrant further investigation.

Additionally, national data suggest that a sharp increase in BMI among Canadian adults was evident within the first year of the pandemic, with the rate of increase in obesity continuing through 2023 (Anderson et al., 2025), which is beyond the observation window of our study.

### Strengths and limitations

This large, population-based study of over 1.1 million pregnancies in Canada’s most populous and diverse province offers near-complete capture of hospital births, minimizing selection bias. The 10-year study period enables assessment of long-term trends, including pandemic-related effects, and detailed sociodemographic information enabling examination of disparities across multiple dimensions.

However, the study also has some limitations. Prepregnancy weight was self-reported for a subset of participants, which may have led to BMI misclassification, as individuals tend to under-report their weight (Dzakpasu et al., 2016; Sharma et al., 2021). This bias can lead to under-estimation of overweight and obesity prevalence. In our study, 717,257 (61.5%) had prepregnancy weight estimated from measured weight at prenatal screening. For the remaining 449,088 (38.5%), weight was obtained from a separate healthcare encounter; whether these values were self-reported or objectively measured, and when they were recorded, is unknown. Weight source was unavailable for 4652 (< 1%) pregnancies. A higher proportion of missing data in prepregnancy BMI was observed in the early study years (2012–2014) and declined substantially by 2016. As the primary BMI increase was concentrated post-2016 when data completeness was highest, the direction of the temporal trend is unlikely to be substantially affected, though early-period estimates should be interpreted with caution. Data on dietary intake, physical activity, and psychosocial stress were unavailable, limiting our ability to fully characterize factors associated with prepregnancy BMI trends. Further, the use of neighbourhood-level socioeconomic status likely resulted in an incomplete picture of socioeconomic disparities, as individual-level variation within neighbourhoods was not captured. In addition, race/ethnicity data were missing for approximately 30% of participants who did not undergo prenatal screening. While we included these participants as a separate category, we could not characterize their racial/ethnic composition. Finally, BMI has inherent limitations as an indicator of adiposity, as it does not differentiate between fat and lean mass or capture fat distribution (Rubino et al., 2025). BMI alone is not a direct measure of health and disease risk at the individual level, and clinical decision-making should consider an individual’s overall health and other risk factors beyond just BMI (Rubino et al., 2025). Despite the limitations of BMI, it is currently incorporated into gestational weight gain guidelines from the Institute of Medicine, with specific weight gain ranges recommended for each BMI group. Until alternative measures are validated for use in pregnancy care, BMI continues to serve as a metric for population surveillance and clinical guidance, though its limitations should be considered when interpreting trends and individual assessments.

## Conclusion

This large population-based study demonstrates significant increases in prepregnancy BMI in Ontario between 2012 and 2022, with acceleration from 2016 to 2017 onward. The shift toward higher BMI categories was accompanied by substantial disparities across socioeconomic strata. While BMI is an imperfect measure that does not capture individual health or the complex factors influencing body weight, these population-level trends have important implications for maternal health. Addressing rising prepregnancy BMI will require moving beyond individual behaviour change, which is challenging and often unsustainable without supportive environments. Effective public health responses will require sustained commitment to health equity and investment in preconception health infrastructure and upstream prevention strategies to improve pregnancy outcomes and reduce health disparities.

## Contributions to knowledge

What does this study add to existing knowledge?

- This study provides Canadian population-level data on prepregnancy BMI trends spanning the COVID-19 pandemic period, filling a critical surveillance gap.
- Mean prepregnancy BMI increased over the study period, with the distribution shifting toward higher weight categories.
- The study reveals disparities across socioeconomic groups, with individuals in the lowest neighbourhood socioeconomic quintiles having the highest BMI and those in the highest quintiles having the lowest BMI.

What are the key implications for public health interventions, practice, or policy?

- Healthcare systems must prepare for the increasing burden of obesity-related pregnancy complications through enhanced capacity and specialized resources.
- Marked disparities necessitate equity-focused interventions addressing structural determinants beyond individual behaviour change.
- Public health strategies should adopt life-course approaches supporting healthy weight across reproductive years.
- Ongoing surveillance is required to monitor whether interventions are reversing trends.

## Data Availability

The data used in this study are securely maintained by the prescribed registry, BORN Ontario. Due to privacy regulations governing personal health information, these data cannot be made publicly available. Requests for access to BORN data should be directed to BORN Ontario (Science@BORNOntario.ca).

## Appendix A

**Table 3:** Trend in prepregnancy BMI from 2012 to 2022 in Ontario, Canada, *N* = 1,170,997

Measure	2012–2013 (*n* = 107,457)	2013–2014 (*n* = 113,350)	2014–2015 (*n* = 115,375)	2015–2016 (*n* = 118,943)	2016–2017 (*n* = 123,328)	2017–2018 (*n* = 121,714)	2018–2019 (*n* = 120,177)	2019–2020 (*n* = 119,566)	2020–2021 (*n* = 115,034)	2021–2022 (*n* = 116,053)	All years (*n* = 1,170,997)
Prepregnancy BMI (kg), Mean (SD)	25.3 (6.2)	25.3 (6.1)	25.3 (6.1)	25.3 (6.0)	25.3 (6.0)	25.6 (6.2)	25.6 (6.2)	25.7 (6.2)	25.8 (6.2)	26.0 (6.3)	25.5 (6.2)
BMI categories, *N* (%)											
Underweight	9,262 (8.6)	9,619 (8.5)	10,078 (8.7)	10,011 (8.4)	10,574 (8.6)	9,853 (8.1)	9,386 (7.8)	9,023 (7.5)	8,256 (7.2)	7,741 (6.7)	93,803 (8.0)
Normal weight	53,904 (50.2)	56,842 (50.1)	57,480 (49.8)	59,051 (49.6)	60,770 (49.3)	58,600 (48.1)	57,341 (47.7)	56,455 (47.2)	53,770 (46.7)	53,332 (46.0)	567,545 (48.5)
Overweight	21,955 (20.4)	22,967 (20.3)	23,525 (20.4)	24,423 (20.5)	25,394 (20.6)	25,612 (21.0)	25,713 (21.4)	25,745 (21.5)	25,282 (22.0)	25,972 (22.4)	246,588 (21.1)
Obesity class I	14,541 (13.5)	15,576 (13.7)	15,678 (13.6)	16,871 (14.2)	17,469 (14.2)	17,813 (14.6)	17,943 (14.9)	18,529 (15.5)	18,038 (15.7)	18,827 (16.2)	171,285 (14.6)
Obesity class II	3,957 (3.7)	4,415 (3.9)	4,547 (3.9)	4,581 (3.9)	5,010 (4.1)	5,165 (4.2)	5,218 (4.3)	5,122 (4.3)	5,129 (4.5)	5,317 (4.6)	48,461 (4.1)
Obesity class III	3,838 (3.6)	3,931 (3.5)	4,067 (3.5)	4,006 (3.4)	4,111 (3.3)	4,671 (3.8)	4,576 (3.8)	4,692 (3.9)	4,559 (4.0)	4,864 (4.2)	43,315 (3.7)

**Table 4:** Joinpoint regression analysis of mean prepregnancy BMI trends, 2012–2021

Segment	Time period	AAC^a^	95% CI	P-value
1	2012–2016	0.016	-0.035, 0.067	0.49
2	2016–2021	0.125	0.088, 0.162	< 0.001

Joinpoint: 2016 (95% CI: 2014–2019) Slope change at joinpoint: 0.109 units/year (P = 0.010)

^a^*AAC*, Average Annual Change in BMI units per year Model selection: 1-joinpoint model (BIC = 2.93) was superior to the linear model (BIC = 3.73) and 2-joinpoint model (BIC = 3.33)

**Table 5:** Regression model of the association between each year (compared to 2012-2013) and prepregnancy BMI

Variable	2013–2014	2014–2015	2015–2016	2016–2017	2017–2018	2018–2019	2019–2020	2020–2021	2021–2022
Model 1 (Unadjusted) β coefficient (95% CI)									
Mean BMI	0.05 (0.01–0.09)	0.19 (0.16–0.23)	0.32 (0.28–0.35)	0.53 (0.49–0.57)	0.84 (0.81–0.88)	1.05 (1.01–1.08)	1.23 (1.19–1.27)	1.46 (1.42–1.50)	1.79 (1.75–1.83)
Model 2 (Partially Adjusted) β coefficient (95% CI)									
Mean BMI	0.01 (-0.03–0.05)	0.05 (0.02–0.09)	0.06 (0.03–0.10)	0.18 (0.14–0.22)	0.39 (0.35–0.43)	0.51 (0.47–0.55)	0.60 (0.56–0.64)	0.74 (0.69–0.78)	0.97 (0.93–1.01)
Model 3 (Fully Adjusted) β coefficient (95% CI)									
Mean BMI	0.01 (-0.03–0.05)	0.04 (0.00–0.08)	0.05 (0.02–0.09)	0.15 (0.11–0.18)	0.34 (0.30–0.38)	0.44 (0.40–0.48)	0.52 (0.48–0.56)	0.65 (0.61–0.69)	0.86 (0.82–0.91)
Model 1 (Unadjusted) Odds ratio (95% CI)									
Underweight	0.98 (0.96–1.02)	1.02 (0.99–1.05)	0.99 (0.96–1.02)	1.01 (0.98–1.04)	0.98 (0.95–1.01)	0.95 (0.92–0.98)	0.93 (0.90–0.96)	0.89 (0.87–0.92)	0.85 (0.82–0.87)
Normal weight (ref)	—	—	—	—	—	—	—	—	—
Overweight	0.99 (0.97–1.01)	1.01 (0.98–1.03)	1.02 (0.99–1.04)	1.03 (1.00–1.05)	1.07 (1.05–1.10)	1.10 (1.08–1.13)	1.12 (1.10–1.14)	1.15 (1.13–1.18)	1.20 (1.17–1.22)
Obesity class I	1.02 (0.99–1.04)	1.01 (0.99–1.04)	1.06 (1.03–1.09)	1.07 (1.04–1.09)	1.13 (1.10–1.15)	1.16 (1.13–1.19)	1.22 (1.19–1.25)	1.24 (1.21–1.28)	1.31 (1.28–1.34)
Obesity class II	1.06 (1.01–1.11)	1.08 (1.03–1.13)	1.06 (1.01–1.10)	1.12 (1.08–1.17)	1.20 (1.15–1.25)	1.24 (1.19–1.29)	1.24 (1.18–1.29)	1.30 (1.25–1.36)	1.36 (1.30–1.42)
Obesity class III	0.97 (0.93–1.02)	0.99 (0.95–1.04)	0.95 (0.91–1.00)	0.95 (0.91–0.99)	1.12 (1.07–1.17)	1.12 (1.07–1.17)	1.17 (1.12–1.22)	1.19 (1.14–1.25)	1.28 (1.23–1.34)
Model 2 (Partially Adjusted) Odds ratio (95% CI)									
Underweight	0.98 (0.95–1.01)	1.01 (0.98–1.04)	0.99 (0.96–1.02)	1.02 (0.99–1.05)	0.99 (0.96–1.02)	0.97 (0.94–1.00)	0.95 (0.92–0.98)	0.91 (0.88–0.94)	0.87 (0.85–0.90)
Normal weight (ref)	—								
Overweight	0.99 (0.97–1.01)	1.00 (0.98–1.02)	1.01 (0.99–1.03)	1.02 (1.00–1.04)	1.06 (1.04–1.09)	1.10 (1.07–1.12)	1.12 (1.09–1.14)	1.16 (1.13–1.18)	1.20 (1.17–1.22)
Obesity class I	1.01 (0.99–1.04)	1.01 (0.98–1.03)	1.05 (1.03–1.08)	1.06 (1.04–1.09)	1.12 (1.09–1.15)	1.16 (1.13–1.19)	1.23 (1.19–1.26)	1.26 (1.23–1.30)	1.33 (1.30–1.37)
Obesity class II	1.05 (1.00–1.10)	1.07 (1.02–1.11)	1.05 (1.00–1.09)	1.12 (1.07–1.17)	1.19 (1.14–1.25)	1.24 (1.19–1.30)	1.25 (1.20–1.30)	1.33 (1.28–1.39)	1.39 (1.33–1.45)
Obesity class III	0.98 (0.93–1.02)	1.00 (0.95–1.04)	0.96 (0.92–1.00)	0.97 (0.92–1.01)	1.13 (1.09–1.19)	1.15 (1.10–1.20)	1.21 (1.16–1.26)	1.26 (1.20–1.31)	1.36 (1.30–1.42)
Model 3 (Fully Adjusted) Odds ratio (95% CI)									
Underweight	0.98 (0.95–1.01)	1.01 (0.98–1.04)	0.99 (0.96–1.02)	1.02 (0.99–1.06)	1.00 (0.97–1.03)	0.98 (0.95–1.01)	0.96 (0.93–0.99)	0.92 (0.89–0.95)	0.89 (0.86–0.92)
Normal weight (ref)	—								
Overweight	0.99 (0.97–1.01)	1.00 (0.98–1.02)	1.01 (0.99–1.03)	1.01 (0.99–1.04)	1.05 (1.03–1.08)	1.08 (1.06–1.11)	1.10 (1.08–1.12)	1.14 (1.12–1.17)	1.18 (1.15–1.21)
Obesity class I	1.01 (0.99–1.04)	1.00 (0.97–1.02)	1.05 (1.02–1.07)	1.05 (1.02–1.07)	1.10 (1.07–1.13)	1.14 (1.11–1.17)	1.19 (1.16–1.22)	1.23 (1.20–1.26)	1.29 (1.26–1.33)
Obesity class II	1.05 (1.00–1.10)	1.06 (1.01–1.11)	1.04 (1.00–1.09)	1.10 (1.05–1.15)	1.16 (1.11–1.21)	1.20 (1.15–1.26)	1.19 (1.14–1.25)	1.28 (1.22–1.34)	1.33 (1.28–1.39)
Obesity class III	0.98 (0.93–1.02)	0.99 (0.95–1.04)	0.95 (0.91–1.00)	0.94 (0.90–0.99)	1.10 (1.05–1.15)	1.11 (1.06–1.16)	1.14 (1.09–1.20)	1.19 (1.14–1.25)	1.28 (1.22–1.34)

Partially adjusted model includes maternal age, race/ethnicity, parity, and neighborhood-level income quintile, education quintile, and material deprivation quintile. Fully adjusted for maternal age, race/ethnicity, parity, and neighborhood-level income quintile, education quintile, and material deprivation quintile, prepregnancy medical composite (prepregnancy hypertension, cardiovascular disease, asthma, or pulmonary disease), prepregnancy diabetes, and smoking status

**Table 6:** Comparison of sociodemographic and clinical characteristics between excluded and included individuals

Characteristics	Included (*n* = 1,170,997)	Excluded^1^ (*n* = 156,398)	SMD
	*n* (%)		
Maternal age (years)			0.09
< 18	5552 (0.5)	1057 (0.7)	
18–24	131,750 (11.3)	18,873 (12.1)	
25–29	312,832 (26.7)	38,790 (24.8)	
30–34	438,385 (37.5)	55,973 (35.8)	
35–39	232,097 (19.8)	32,651 (20.9)	
≥ 40	49,874 (4.3)	8906 (5.7)	
Race/ethnicity			0.322
Asian	215,753 (18.6)	31,057 (20.1)	
Black	53,786 (4.6)	8476 (5.5)	
White	490,034 (42.3)	42,750 (27.7)	
Other^a^	48,088 (4.1)	7444 (4.8)	
Missing due to no PSO screening	351,864 (30.3)	64,543 (41.8)	
Education quintiles at dissemination level			
Q1 (Lowest)	223,103 (19.5)	29,874 (19.7)	0.105
Q2	240,198 (21.0)	30,053 (19.8)	
Q3	208,020 (18.2)	25,407 (16.8)	
Q4	263,848 (23.1)	32,383 (21.4)	
Q5 (highest)	209,385 (18.3)	33,833 (22.3)	
Material deprivation quintiles			
Q1 (Least)	202,471 (17.8)	25,484 (17.1)	0.063
Q2	240,500 (21.1)	29,012 (19.4)	
Q3	232,928 (20.5)	30,332 (20.3)	
Q4	212,257 (18.6)	28,293 (18.9)	
Q5 (Most)	250,735 (22.0)	36,258 (24.3)	
Median household income quintile			
Q1 (Lowest)	242,708 (21.3)	33,265 (22.1)	0.052
Q2	218,157 (19.2)	29,453 (19.6)	
Q3	211,728 (18.6)	26,389 (17.5)	
Q4	242,325 (21.3)	30,040 (19.9)	
Q5 (highest)	222,958 (19.6)	31,506 (20.9)	
Parity			
Nulliparous	511720 (44.0)	61950 (41.0)	0.106
Primiparous	409104 (35.1)	50986 (33.7)	
Multiparous	243202 (20.9)	38299 (25.3)	
Smoking status			
No	1,045,288 (91.0)	125,309 (90.3)	0.022
Yes	103,918 (9.0)	13,447 (9.7)	
Prepregnancy diabetes			
No	1,148,226 (99.0)	143,065 (98.7)	0.022
Yes	11,901 (1.0)	1825 (1.3)	
Pre-existing medical condition^b^			
No	1,068,296 (94.6)	123,606 (95.5)	0.045
Yes	61,396 (5.4)	5767 (4.5)	
Year of childbirth			0.304
2012	107,457 (9.2)	26,745 (17.1)	
2013	113,350 (9.7)	19,430 (12.4)	
2014	115,375 (9.9)	17,483 (11.2)	
2015	118,943 (10.2)	14,017 (9.0)	
2016	123,328 (10.5)	10,320 (6.6)	
2017	121,714 (10.4)	11,906 (7.6)	
2018	120,177 (10.3)	13,400 (8.6)	
2019	119,566 (10.2)	13,829 (8.8)	
2020	115,034 (9.8)	13,992 (8.9)	
2021	116,053 (9.9)	15,276 (9.8)	

*SMD*, standardized mean differences; *PSO*, Prenatal Screening Ontario; *BMI*, body mass index

^1^Excluded records were individuals with missing value for prepregnancy BMI or implausible values

^a^Including mixed ethnicity, unknown, and other groups

^b^A composite of either prepregnancy hypertension, cardiovascular disease, asthma, or pulmonary disease

Percentages represent column proportions within each group

Within-year proportion missing (excluded/[included + excluded]): 2012–13: 19.9%; 2013–14: 14.6%; 2014–15: 13.2%; 2015–16: 10.5%; 2016–17: 7.7%; 2017–18: 8.9%; 2018–19: 10.0%; 2019–20: 10.4%; 2020–21: 10.8%; 2021–22: 11.6%

**Table 7:** Comparison of sociodemographic and clinical characteristics between excluded and included individuals (including missing data rows)

Characteristics	Included (*n* = 1,170,997)	Excluded^1^ (*n* = 156,398)		SMD
	*n* (%)			
Maternal age (years)				
< 18	5552 (0.5)	1057 (0.7)		
18–24	131,750 (11.3)	18,873 (12.1)		
25–29	312,832 (26.7)	38,790 (24.8)		0.092
30–34	438,385 (37.4)	55,973 (35.8)		
35–39	232,097 (19.8)	32,651 (20.9)		
≥ 40	49,874 (4.3)	8906 (5.7)		
Missing	507 (0.0)	148 (0.1)		
Race/ethnicity				0.322
Asian	215,753 (18.4)	31,057 (19.9)		
Black	53,786 (4.6)	8476 (5.4)		
White	490,034 (41.8)	42,750 (27.3)		
Other^a^	48,088 (4.1)	7444 (4.8)		
Missing due to no PSO screening	351,864 (30.0)	64,543 (41.3)		
Missing	11,472 (1.0)	2128 (1.4)		
Education quintiles at dissemination level				
Q1 (Lowest)	223,103 (19.1)	29,874 (19.1)		0.116
Q2	240,198 (20.5)	30,053 (19.2)		
Q3	208,020 (17.8)	25,407 (16.2)		
Q4	263,848 (22.5)	32,383 (20.7)		
Q5 (highest)	209,385 (17.9)	33,833 (21.6)		
Missing	26,443 (2.3)	4848 (3.1)		
Material deprivation quintiles				
Q1 (Least)	202,471 (17.3)	25,484 (16.3)		0.112
Q2	240,500 (20.5)	29,012 (18.6)		
Q3	232,928 (19.9)	30,332 (19.4)		
Q4	212,257 (18.1)	28,293 (18.1)		
Q5 (Most)	250,735 (21.4)	36,258 (23.2)		
Missing	32,106 (2.7)	7019 (4.5)		
Median household income quintile				
Q1 (Lowest)	242,708 (20.7)	33,265 (21.3)		0.07
Q2	218,157 (18.6)	29,453 (18.8)		
Q3	211,728 (18.1)	26,389 (16.9)		
Q4	242,325 (20.7)	30,040 (19.2)		
Q5 (highest)	222,958 (19.0)	31,506 (20.1)		
Missing	33,121 (2.8)	5745 (3.7)		
Parity				
Nulliparous	511,720 (43.7)	61,950 (39.6)		0.223
Primiparous	409,104 (34.9)	50,986 (32.6)		
Multiparous	243,202 (20.8)	38,299 (24.5)		
Missing	6971 (0.6)	5163 (3.3)		
Smoking status				
No	1,045,288 (89.3)	125,309 (80.1)		0.388
Yes	103,918 (8.9)	13,447 (8.6)		
Missing	21,791 (1.9)	17,642 (11.3)		
Prepregnancy diabetes				
No	1,148,226 (98.1)	143,065 (91.5)		0.328
Yes	11,901 (1.0)	1825 (1.2)		
Missing	10,870 (0.9)	11,508 (7.4)		
Pre-existing medical condition^b^				
No	1,068,296 (91.2)	123,606 (79.0)		0.464
Yes	61,396 (5.2)	5767 (3.7)		
Missing	41,305 (3.5)	27,025 (17.3)		
Year of childbirth				0.304
2012	107,457 (9.2)	26,745 (17.1)		
2013	113,350 (9.7)	19,430 (12.4)		
2014	115,375 (9.9)	17,483 (11.2)		
2015	118,943 (10.2)	14,017 (9.0)		
2016	123,328 (10.5)	10,320 (6.6)		
2017	121,714 (10.4)	11,906 (7.6)		
2018	120,177 (10.3)	13,400 (8.6)		
2019	119,566 (10.2)	13,829 (8.8)		
2020	115,034 (9.8)	13,992 (8.9)		
2021	116,053 (9.9)	15,276 (9.8)		

*SMD*, standardized mean differences; *PSO,* Prenatal Screening Ontario, *BMI,* body mass index

^1^Excluded records were individuals with missing value for prepregnancy BMI or implausible values

^a^Including mixed ethnicity, unknown, and other groups

^b^A composite of either prepregnancy hypertension, cardiovascular disease, asthma,  or pulmonary disease

Percentages represent column proportions within each group

## Code availability

Available upon request and approval by BORN Ontario.
